# MCPIP-1 Restricts Inflammation via Promoting Apoptosis of Neutrophils

**DOI:** 10.3389/fimmu.2021.627922

**Published:** 2021-02-26

**Authors:** Ewelina Dobosz, Marta Wadowska, Marta Kaminska, Mateusz Wilamowski, Mohsen Honarpisheh, Danuta Bryzek, Jan Potempa, Jolanta Jura, Maciej Lech, Joanna Koziel

**Affiliations:** ^1^Department of Microbiology, Faculty of Biochemistry, Biophysics and Biotechnology of Jagiellonian University, Krakow, Poland; ^2^Department of General Biochemistry, Faculty of Biochemistry, Biophysics and Biotechnology of Jagiellonian University, Krakow, Poland; ^3^Ludwig-Maximilians University Hospital, Medizinische Klinik und Poliklinik IV, Ludwig-Maximilians University, Munich, Germany; ^4^Department of Oral Immunity and Infectious Diseases, University of Louisville School of Dentistry, University of Louisville, Louisville, KY, United States

**Keywords:** MCPIP-1, apoptosis, neutrophils, miRNA, inflammation, GM-CSF

## Abstract

Monocyte chemoattractant protein-induced protein-1 (MCPIP-1) is a potent inhibitor of inflammatory response to pathogens. Acting as endonuclease against transcripts of inflammatory cytokines or transcription factors MCPIP-1 can significantly reduce the cytokine storm, thus limiting the tissue damage. As the adequate resolution of inflammation depends also on the efficient clearance of accumulated neutrophils, we focused on the role of MCPIP-1 in apoptosis and retention of neutrophils. We used peritoneal neutrophils from cell-specific MCPIP-1 knockout mice and showed prolonged survival of these cells. Moreover, we confirmed that MCPIP-1-dependent degradation of transcripts of antiapoptotic genes, including *BCL3, BCL2A1, BCL2L1*, and for the first time *MCL-1*, serves as an early event in spontaneous apoptosis of primary neutrophils. Additionally, we identified previously unknown miRNAs as potential binding partners to the *MCPIP-1* transcript and their regulation suggest a role in MCPIP-1 half-life and translation. These phenomena may play a role as a molecular switch that balances the MCPIP-1-dependent apoptosis. Besides that, we determined these particular miRNAs as integral components of the GM-CSF-MCPIP-1 axis. Taken together, we identified the novel anti-inflammatory role of MCPIP-1 as a regulator of accumulation and survival of neutrophils that simultaneously promotes an adequate resolution of inflammation.

## Introduction

Monocyte chemoattractant protein-induced protein-1 (MCPIP-1), also called Regnase-1, is a potent negative regulator of the inflammatory response. MCPIP-1 is a multidomain protein, composed of ubiquitin-binding and RNase-like PIN domains. Both domains are crucial determinants of the anti-inflammatory function of MCPIP-1. Its involvement in deubiquitination process provides inhibition of LPS- and IL-1-induced NF-κB signaling pathway, whereas RNase activity controls mRNA level for proinflammatory molecules, like IL-6, IL-1β, or IL-8 (1–5). Moreover, MCPIP-1 seems to play a role in processes of cell differentiation, osteoclastogenesis, angiogenesis, adipogenesis, and cell death (6–9). Initially, MCPIP-1 was described as a new transcription factor for an apoptotic gene family, which contributes to ischemic heart disease (10). MCPIP-1-deficient mice display restrained apoptosis and enhanced proliferation of epithelial cells due to the effects of MCPIP-1 on the mTOR pathway and purine metabolism (11). Moreover, studies on macrophages revealed that MCPIP-1 suppresses stress granule formation, thus determining cell apoptosis as a result of pro-apoptotic transcripts release (12). Consequently, another study demonstrated that several anti-apoptotic gene transcripts can serve as substrates for MCPIP-1 in breast cancer cells (13). The involvement of MCPIP-1 in cell death regulation was confirmed by delayed death of Mcpip-1-deficient CD8^+^ T cells infiltrating tumor (14). However, the role of MCPIP-1 in neutrophils' responses and survival, has not been examined yet.

Neutrophils represent an early, crucial, and potent line of defense during infection and injury. As the components of cell granules are highly toxic, the process of neutrophils recruitment, activation, death, and clearance must be strictly controlled. The abnormalities in neutrophil function lead to the development of persisting inflammation and progressive tissue damage (15). Their responses play a key role in the restoration of homeostasis and etiology of septic and aseptic inflammatory conditions, such as inflammatory bowel disease, periodontitis as well as sterile inflammation such as COPD or ischemia-reperfusion injury (16–18).

Thus, in the presented study, we investigated the engagement of MCPIP-1 in the regulation of programmed cell death of neutrophils. We showed that MCPIP-1 regulates the survival of neutrophils, by orchestrating the expression of pro- and anti-apoptotic proteins. Additionally, we identified miRNAs responsible for the restriction of MCPIP-1 translation and classified these miRNAs as integral components of the GM-CSF-MCPIP-1 axis. Taken together, we identified the novel role of MCPIP-1 as a factor controlling the homeostasis of neutrophils.

## Materials and Methods

### Mice Experiments

Specific inactivation of the *Mcpip-1* gene in myelomonocytic cells was achieved by crossing the floxed MCPIP-1 mice (obtained from Prof. Mingui Fu) with Lys-M-Cre mice (Jackson Laboratories) as described previously (19). Animals were housed in ventilated cages under SPF conditions with a 14/10 h light/dark cycle and fed *ad libitum*. For experiments, we used 8–10 weeks old female mice. For aseptic and septic peritonitis, mice were intraperitoneally injected with 1 ml of 4% sterile thioglycolate (Fluka, USA) or with 10 mg/kg of LPS from *E. coli* for 3 h. Peritoneal exudates were collected under anesthesia by washing the peritoneal cavity with 5 ml of PBS (Gibco/Thermo Fisher Scientific, USA). After centrifugation at 280 × g for 5 min, red blood cells were lysed with water, and peritoneal neutrophils were resuspended in DMEM without phenol red (Gibco/Thermo Fisher Scientific) and counted. All experiments were approved by the Institutional Animal Care and Use Committees (Jagiellonian University, Krakow, Poland; permit number: 228/2017; 92/2020).

### Chemokine Assay

The level of murine MCP-1, KC, and RANTES in peritoneal fluid of thioglycolate injected mice was determined using commercially available BD™ Cytometric Bead Array Chemokine Flex Sets (BD Biosciences, USA). Chemokine concentration was determined using FACScan flow cytometer and analyzed with CellQuest software.

### Morphological Estimation of Apoptosis

Murine neutrophils isolated from peritoneum were cultured in DMEM for 7 h, seeded using Cytospin3 (Shandon, UK) and stained with May-Grünwald-Giemsa (Bio Optica Milano, Italy). On each slide, at least 250 cells were counted using × 60 objective with immersion on Evos fluorescence microscope (Thermo Fisher Scientific). Apoptotic cells were enumerated based on an alternated morphology of cell nuclei (20).

### Analysis of Phosphatidylserine (PS) Exposure

Exposure of PS was determined using ApoFlowEx®-FITC kit (Exbio, Czech Republic) according to manufacturer's instructions. Briefly, murine and human cells were cultivated in 1 mln/ml density in the DMEM or RPMI supplemented with 5% FBS, respectively and after the indicated time (1 or 7 h) cells were stained with annexin V-FITC conjugates and PI (100 μg/ml each) for 15 min in the dark. Then cells were centrifuged (280 × g, 5 min), collected with FACScan flow cytometer (Becton Dickinson) and analyzed using CellQuest software.

### Analysis of Caspase-3 Activity

Caspase-3 activity was quantified using Ac-DEVD-AMC peptide (Sigma) as described (21). Murine neutrophils were cultivated for 7 h in DMEM, then centrifuged (280 × g, 5 min), washed once with ice-cold PBS and resuspended in lysis buffer (50 mM Tris, pH 7.5, 150 mM NaCl, 1% NP-40, 0.5% deoxycholic acid, 0.1% SDS). After 20 min of incubation on ice, lysates were centrifuged (16,000 × g, 15 min, 4°C) and the protein level was determined in collected supernatant using the BCA method. Equal amount of protein extracts were transferred into reaction buffer (40 mM Pipes, 20% sucrose, 200 mM NaCl, 0.2% CHAPS, 2 mM EDTA) with addition of 0.1 mM Ac-DEVD-AMC substrate (Enzo Life Sciences, USA). The cleavage of the fluorogenic peptide was monitored up to 30 min by AMC liberation and increase of fluorescence (λ_ex_ = 350 nm, λ_em_ = 460 nm) using a FlexStation3 (Molecular Devices). We showed the endpoint values of fluorescence measured 15 min post-reaction's start.

### Isolation of Human Neutrophils

Peripheral blood from de-identified human donors was obtained from the Red Cross (Krakow, Poland). Neutrophils were isolated within 1 h post bleeding using lymphocyte separation medium (Pan Biotech, Germany) as described (22). Neutrophils and erythrocytes were collected as the high-density fraction and separated after 30 min of incubation with 1% polyvinyl alcohol (POCH, Poland). Neutrophils were collected from the upper layer, and after centrifugation (280 × g, 10 min), the residual erythrocytes were removed by lysis in water. The purity of PMN preparations was examined using FITC anti-human CD15 (SSEA-1) antibody and was estimated in a range between 94 and 95%. Neutrophils were resuspended in RPMI 1640 with 5% of FBS (Gibco/Thermo Fisher Scientific) and subjected to further analysis.

### Incubation of Neutrophils With Anti-apoptotic Factors

Human neutrophils were treated with: 20 ng/ml GM-CSF (R&D Systems, USA), 10 ng/ml G-CSF (Amgen, USA), 200 ng/ml IL-8 (R&D Systems) or 2 μg/ml C5a (Complement Technology, USA) in RPMI 1640 supplemented with 5% FBS. Cells were subjected to gene expression, Western blot and cell death analysis. For neutralization of GM-CSF activity, cells were preincubated for 15 min with 1 μg/ml neutralizing anti-GM-CSF antibodies (R&D Systems) or isotypic antibodies (Santa Cruz Biotechnology, USA).

### Quantitative Reverse Transcription PCR

Neutrophils cultivated for an indicated time were centrifuged (280 × g, 10 min) and lysed for estimation of transcripts expression. Total RNA was isolated using TRIzol Reagent (Thermo Fisher Scientific) and quantification of mRNA was performed as described (5). Reverse transcription was conducted using a High-Capacity cDNA Reverse Transcription Kit (Applied Biosystems, USA). cDNA synthesis reaction was performed using 300 ng of RNA and random primers in a total volume of 20 μl, according to the manufacturer's recommendation. Quantitative PCR reaction (qPCR) was performed with SYBR Green dye in final reaction volume 15 μl, containing 1 μl of cDNA sample, 0.3 μM of each primer and GoTaq® qPCR MasterMix (Promega, USA). Primer sequences and reaction conditions are listed in Table 1. After 5 min of initial denaturation in 95°C, reactions were carried out for 40 cycles, which was followed by a final elongation step at 72°C for 10 min. All reactions were performed in duplicates. For verification of the quality of PCR products, melting curves were generated. The gene expression was calculated as the difference in the cycle threshold (ΔC_t_) between the target gene and housekeeping elongation factor 2: *EF2* for human or *Ef2* for murine gene; ΔΔC_t_ was the difference between the ΔC_t_ values of the tested and control sample. The expression of the target genes was calculated as 2^−ΔCt^–designated on graphs as “gene expression” and 2^−ΔΔCt^–designated on figures as “relative gene expression” (23).

**Table 1:** List of oligonucleotides used in the studies.

**Oligonucleotide**	**Sequence**	**Temperature program**
		**1 —denaturation,** **2 —annealing,** **3 —extension**
*m_Ef2_R*	5′-TCAGCACACTGGCATAGAGGC-3′	1.95°C, 20 s2. 6 2°C, 20 s 3. 72°C, 30 s
*m_Ef2_F*	5′-GACATCACCAAGGGTGTGCAG-3′	
*m_Mcpip-1_R*	5′-CAGCCGCTCCTCGATGAAGC-3′	
*m_Mcpip-1_F*	5′-CAGCCTCGACCAGATGTGCC-3′	
*m_Bid_R*	5′-CACTCAAGCTGAACGCAGAG-3′	1.95°C, 30 s 2. 57°C, 30 s 3. 72°C, 30 s
*m_Bid_F*	5′-TGTGAGGAACTTGGTTAGAAACG-3′	
*m_Bcl2a1_R*	5′-TGCTGCATTGTTCCCGTAGA-3′	
*m_Bcl2a1_F*	5′-GCATCGTGGCCTTTTTCTCC-3′	
*m_Mcl-1_R*	5′-TAAGGACGAAACGGGACTGG-3′	
*m_Mcl-1_F*	5′-TAAGGACGAAACGGGACTGG-3′	
*m_Bim_R*	5′-CCTGTGCAATCCGTATCTCCG-3′	1.95°C, 30 s 2. 64°C, 30 s 3. 72°C, 30 s
*m_Bim_F*	5′-GCCAGGCCTTCAACCACTATC-3′	
*EF2_R*	5′-TTCAGCACACTGGCATAGAGGC-3′	1.95°C, 30 s 2. 62°C, 30 s 3. 72°C, 30 s
*EF2_F*	5′-GACATCACCAAGGGTGTGCAG-3′	
*MCPIP-1_R*	5′-TCCAGGCTGCACTGCTCACTC-3′	
*MCPIP-1_F*	5′-GGAAGCAGCCGTGTCCCTATG-3′	
*IL-8_R*	5′-TCTCAGCCCTCTTCAAAAACTTCT-3′	1.95°C, 20 s 2. 60°C, 1 min 3. 72°C, 45 s
*IL-8_F*	5′-ATGACTTCCAAGCTGGCCGTGGCT-3′	
*MCL-1_R*	5′-CCAGCTCCTACTCCAGCAA-3′	1.95°C, 20 s 2. 58°C, 1 min 3. 72°C, 1 min
*MCL-1_F*	5′-TAAGGACAAAACGGGACTGG-3′	
*BAK-1_R*	5′-GTCAGGCCATGCTGGTAGAC-3′	1.95°C, 15 s 2. 58°C, 15 s 3. 72°C, 20 s
*BAK-1_F*	5′-CATCAACCGACGCTATGACTC-3′	
*BID_R*	5′-CTTTGGAGGAAGCCAAACAC-3′	
*BID_F*	5′-CCATGGACTGTGAGGTCAAC-3′	
*BAX_R*	5′-TCAGCCCATCTTCTTCCAGA-3′	1.95°C, 15 s 2. 60°C, 30 s 3. 72°C, 30 s
*BAX_F*	5′-GCTGTTGGGCTGGATCCAAG-3′	
*BCL2A1_R*	5′-ACAAAGCCATTTTCCCAGCCT-3′	
*BCL2A1_F*	5′-AAATTGCCCCGGATGTGGAT-3′	
*BCL2L1_R*	5′-ACAAAAGTATCCCAGCCGCC-3′	
*BCL2L1_F*	5′-CTGTGCGTGGAAAGCGTAGA-3′	
*BCL3_R*	5′-ACATTTGCGCGTTCACGTT-3′	
*BCL3_F*	5′-TCGACGCAGTGGACATTAAGAG-3′	
*RELB_R*	5′-GAACATGTTGCTGCCCACAAG-3′	
*RELB_F*	5′-CATCCTGGACCACTTCCTGCC-3′	

### Protein Isolation and Immunoblot Analysis

Cellular extracts were prepared in RIPA buffer supplemented with protease inhibitor cocktail (Roche, Switzerland) and analyzed by immunoblotting as described (5). To obtain the whole cellular extracts, cells were resuspended in RIPA lysis buffer (0.25% Na-deoxycholate, 0.5% Nonidet P-40, 0.05% SDS, 2.5 mM EDTA in PBS) supplemented with a protease inhibitor cocktail (Roche, Switzerland). Immediately after lysis, extracts were frozen in liquid nitrogen and stored in −20°C. The protein concentration was assessed before electrophoresis using a BCA protein assay (Thermo Fisher Scientific). Twelve percent SDS-PAGE gels were used for separation of equal amounts of protein (20 μg per well), which was followed by their electrotransfer onto PVDF membranes (Merck Milipore, USA) in transfer buffer (25 mM Tris, 0.2 M glycine, and 20% methanol) for 1.5 h at 100 V. Nonspecific binding sites were blocked for 2 h in TBST buffer (20 mM Tris, pH 7.5, 0.5 M NaCl, 0.05% Tween 20) containing 5% skimmed milk (BioShop). Rabbit anti-MCPIP-1 (GeneTex, USA), rabbit anti-MCL-1 (Rockland, USA), rat anti-BCL2A1 (Sigma), rabbit anti-GAPDH (Cell Signaling, Netherlands) or mouse anti-β-actin (BD Bioscience) followed by HRP-conjugated secondary antibodies: goat anti-rabbit (Cell Signaling), goat anti-rat (Cell Signaling) and goat anti-mouse (BD Biosciences) were used. Blots were developed using Luminata Crescendo Substrate (Merck Milipore) and visualized using ChemiDoc^TM^ Touch Imaging System (BioRad, USA). Densitometric analyses of Western blots were performed using Image J software. The results are presented as mean values in arbitrary densitometric units, corrected for background intensity, or as increases over the corresponding levels in nonstimulated cells.

### RNA-Protein Immunoprecipitation

Immunoprecipitation procedures were conducted using HeLa cell line as was described previously (5). Evaluation of transcripts which are substrates for MCPIP-1 protein was performed using rabbit anti-MCPIP1 antibodies (GeneTex).

### Determination of microRNA Expression

Estimation of mature microRNA expression was performed using miRCURY LNA^TM^ Universal RT microRNA system (Qiagen, USA) according to the manufacturer's instructions. Shortly, 150 ng of total RNA was used to synthesize the first-strand cDNA using miRCURY LNA^TM^ RT kit, which was followed by qPCR amplification using miRCURY LNA^TM^ SYBR Green PCR kit. MicroRNA expression level was normalized to internal reference gene U6 snRNA. The primer sequences used in procedures were based on commercially available primer sets: miRCURY LNA^TM^ miRNA PCR Assay (Qiagen).

### miRNA-mRNA EMSA Assay

The miRNA oligonucleotide was labeled with Cy5 dye on its 5′ end and the 2′ O-methyl-modified *MCPIP-1* mRNA oligonucleotide was labeled with Atto488 dye on its 5′ end (Sigma). Oligonucleotides were separately heated for 5 min at 80°C, placed on ice to relax RNA secondary structure, mix in binding buffer (100 mM HEPES pH 7.3, 200 mM KCl, 10 mM MgCl_2_, 5% glycerol) and then incubated at 25°C for 25 min (24). The reaction mixtures were separated on a 15% PAGE by native electrophoresis at 4°C, and the resultant mobility shifts were detected with ChemiDoc^TM^ Touch Imaging System (BioRad). All procedures were conducted in RNase free conditions.

### Bioinformatic Analysis

Prediction for 8mer, 7mer, and 6mer seed regions that match miRNA binding sites in the human *MCPIP-1* 3′UTR mRNA was done with TargetScan v7.2 software (25–27). Alignments of miRNA binding sites to human MCPIP-1 3′UTRs and their orthologs were performed in TargetScan v7.2 based on UCSC whole-genome alignments (26). Estimation of minimal free energy of binding miRNA with *MCPIP-1* mRNA was done with RNAhybrid tool (28). Prediction of the secondary structure of the *MCL-1* mRNA (NM_021960.5) was performed using the RNAfold web server from the Vienna RNA website (29). Identification of stem-loop structures in the *MCL-1* mRNA that are potential MCPIP-1 targets was based on a search of the MCPIP-1 consensus binding motif, which is enriched with UAU sequence at the loop site (30).

### Statistical Analysis

Statistical analysis was performed using parametric Student's *t*-tests or one-way factorial analysis of variance (ANOVA) using GraphPad Prism 7.4. Software. The statistical significance were indicated as follows: *p*-value < 0.05 (^*^); *p*-value of < 0.01 (^**^); *p*-value of < 0.001 (^***^); ns for non-significant.

## Results

### Accelerated Influx and Survival of Granulocytes in Inflamed Tissue Is Controlled by MCPIP-1

**Figure 1:**
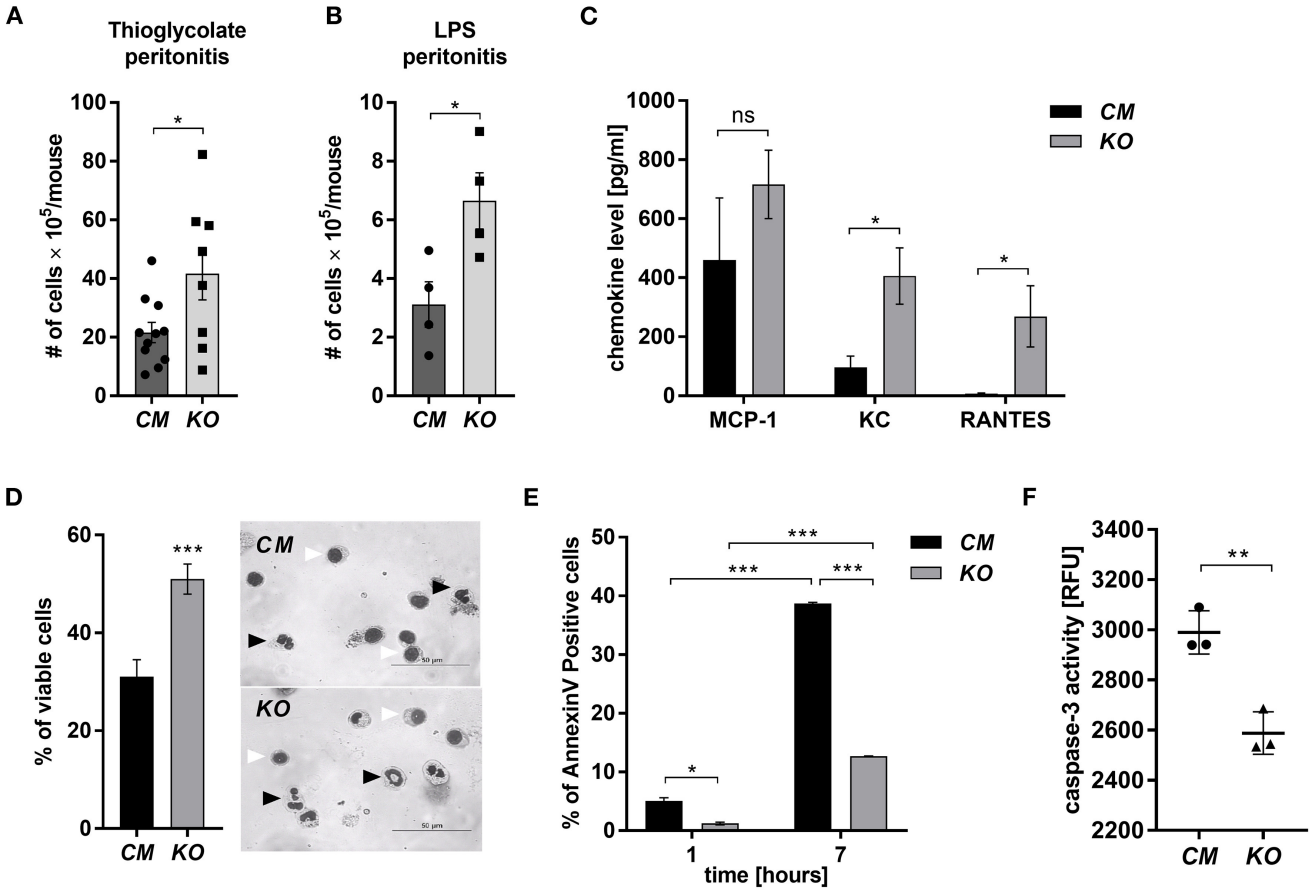
MCPIP-1 affects neutrophils during inflammation. **(A,B)** Accumulation of neutrophils in **(A)** thioglycolate or **(B)** LPS-induced peritonitis; *n* = 4–11 **(C–F)** Neutrophils were isolated from *Control Mutant* (*CM*) and *Mcpip-1-KO* (*KO*) mice 3 h post i.p. injection with thioglycolate. **(C)** The chemokine level in the peritoneal fluid was examined using Chemokine Beads Array; the mean ± SEM; *n* = 4 **(D)** May-Grünwald-Giemsa staining performed 7 h post isolation of neutrophils. Bars represent the percentage of viable PMNs among 250 counted cells; the mean ± SEM (left). Representative images of stained neutrophils were shown (right)—black-head arrows indicate an example of viable cells, white-head arrows indicate apoptotic cells **(E)** Neutrophils were double-stained with Annexin V and PI and subjected to FACS. The representative result of Annexin V positive cells was shown; the mean ± SEM. **(F)** The endpoint values of caspase-3 activity after 15 min of reaction in neutrophils previously cultivated for 7 h. Data show representative RFU changes in time (Relative Fluorescence Unit); the mean ± SEM; *n* = 3. *p*-value of < 0.05 (*); *p*-value of < 0.01 (**); *p*-value of < 0.001 (***); ns for non-significant.

MCPIP-1 is considered a potent negative regulator of inflammation, but its contribution to the granulocytes (PMNs) fate remains unknown. Therefore, we examined its role in the biology of PMNs using transgenic animals in which the *Mcpip-1* gene was selectively silenced in phagocytes (MCPIP1-Flox-M-Lys-CRE mice) (Supplementary Figure 1). Using two different models of inflammation: septic and aseptic peritonitis, we found an increased number of PMNs in the affected tissue in mice with phagocytes-specific *Mcpip-1* deficiency (Figures 1A,B). An enhanced influx of granulocytes was associated with an elevated level of chemokines (KC, RANTES) in the foci of inflammation (Figure 1C). Since the resolution of inflammatory reaction strongly depends on the death of infiltrating PMNs and their proper clearance, we focused our attention on the viability of PMNs *ex vivo*, isolating them from knock-out and wild-type mice. Using Giemsa staining, we found a significantly higher number of cells with properly segmented nuclei in mice lacking the *Mcpip-1* gene (Figure 1D). Reduced apoptosis in MCPIP-1 deficient PMNs was confirmed by elevated exposition of phosphatidylserine on the cell surface in control PMNs (Figure 1E) and significantly higher activation of caspase-3 (Figure 1F). Altogether, we showed that MCPIP-1 is involved in the regulation of cell death of neutrophils *ex vivo*.

### The Regulation of Spontaneous Death of Neutrophils by MCPIP-1

**Figure 2:**
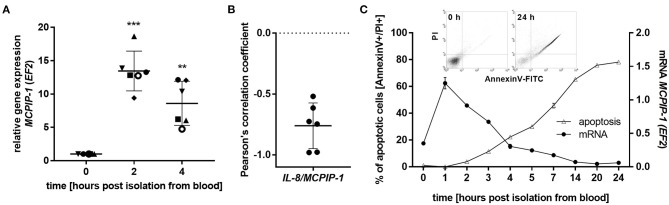
MCPIP-1 is rapidly induced and preceded spontaneous neutrophil apoptosis. **(A–C)** Neutrophils were isolated from human blood and cultivated as indicated. **(A)** *MCPIP-1* transcript level was determined using qPCR. Bars represent the mean relative expression to time 0 (*t*_0_ = 1) ± SD; each symbol indicates different donor **(B)** Pearson's correlation coefficient values were calculated based on *MCPIP-1* and *IL-8* transcript levels. *n* = 6 **(C)** *MCPIP-1* transcript level was determined using qPCR (circle). Cells were double-stained with Annexin V and PI (triangle), and analyzed using flow cytometry. A representative result was shown ± SEM. PI, propidium iodide. *p*-value of < 0.01 (**); *p*-value of < 0.001 (***).

**Figure 3:**
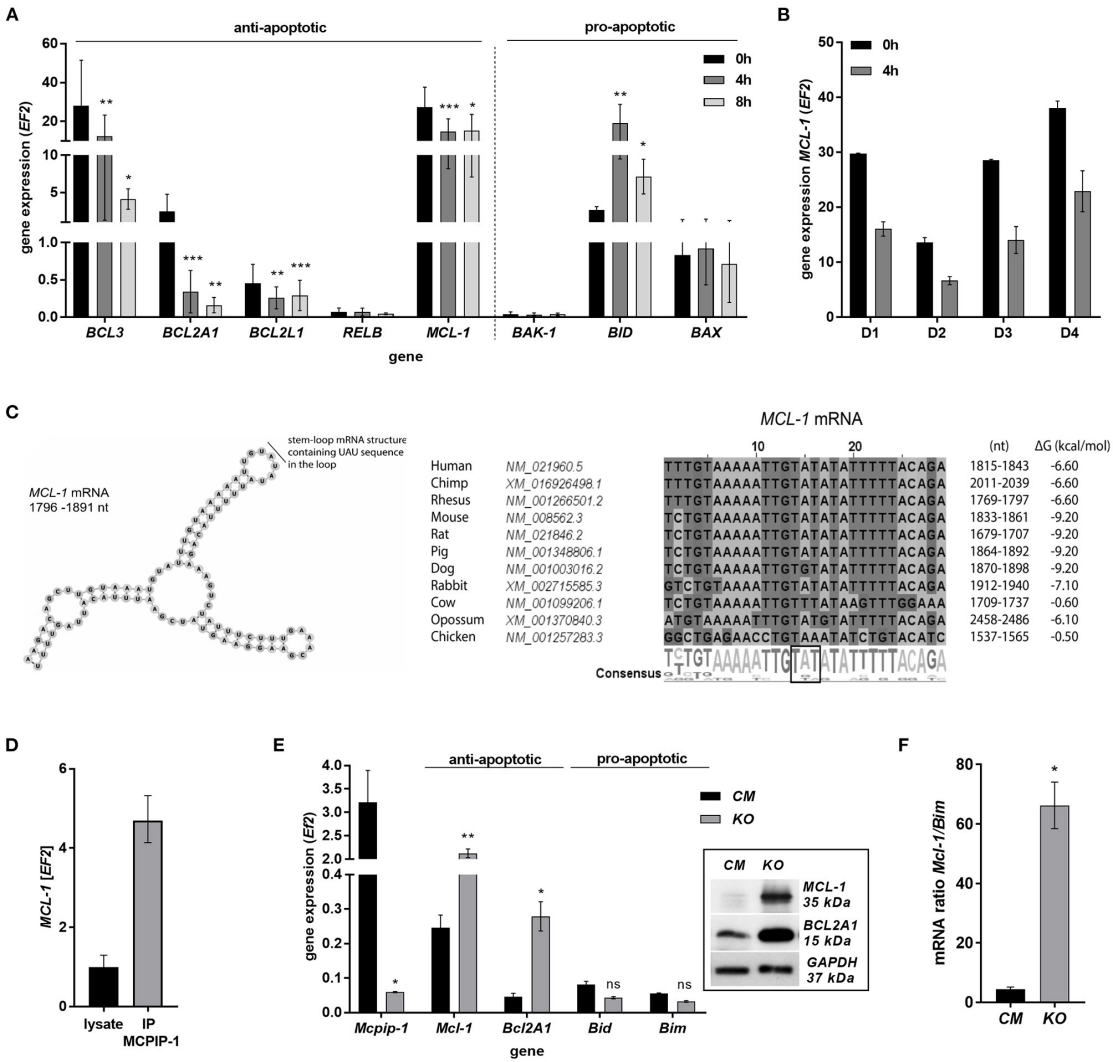
MCPIP-1 induces spontaneous apoptosis of neutrophils by regulation of anti-apoptotic transcripts. **(A,B)** Neutrophils were isolated from human blood and cultivated as indicated. **(A)** Relative expression of apoptotic genes analyzed by qPCR. *n* = 4–7. Bars represent the mean relative expression ± SD. **(B)** Graph shows mean expression of *MCL-1* mRNA in four different donors (D1-4) ± SEM. **(C)** The secondary structure of 3′UTR fragment of the *MCL-1* mRNA from a depicted region between 1,796 and 1,891 nt (left). Evolutionary conserved among species and thermodynamically stable RNA stem-loops secondary structures from 3′UTR transcript of *MCL-1* (right). **(D)** The level of *MCL-1* mRNA in cell lysates before and after immunoprecipitation with an anti-MCPIP-1 antibody. **(E)** Relative mean expression of apoptotic genes in murine neutrophils isolated from peritoneum of *Control Mutant* (*CM*) and *Mcpip-1-KO* (*KO*) mice 3 h post-injection with thioglycolate. *n* = 3–5 animals representative Western blot analysis is presented in insert. **(F)** The graph represents a ratio between expression of *Mcl-1* and *Bim* mRNA in murine neutrophils ± SEM. *p*-value of < 0.05 (*); *p*-value of < 0.01 (**); *p*-value of < 0.001 (***); ns for non-significant.

The finding that MCPIP-1 controls the life-span of mice PMNs *ex vivo* stimulated us to investigate its role in the apoptosis of human neutrophils. Firstly, we correlated the changes in MCPIP-1 expression with spontaneous cell death (31) finding rapid, but transient upregulation of *MCPIP-1* observed within 1–4 h post PMNs isolation from the blood (Figure 2A). The profile of *MCPIP-1* mRNA changes was independent of a donor and correlated negatively with *IL-8* mRNA expression, a cytokine that serves as a substrate for MCPIP-1 RNase (Figure 2B). Further analysis showed that the upregulation of *MCPIP-1* preceded initial symptoms of neutrophils apoptosis (Figure 2C). Therefore, we determined levels of transcripts encoding anti-apoptotic proteins *BCL3, BCL2A1, BCL2L1, RELB*, and *MCL-1*. We found a significant decrease of the corresponding mRNA, except *RELB*, 4 h post neutrophils isolation (Figures 3A,B). At the same time, we observed that the expression of pro-apoptotic genes remains stable (*BAK-1, BAX*) or increases (*BID*) in time (Figure 3A) (32). Among all tested transcripts of anti-apoptotic proteins, *MCL-1* has not been identified as a substrate of MCPIP-1 yet. Our bioinformatics analysis confirmed that a fragment of 3′UTR of the *MCL-1* mRNA, between 1,815 and 1,843 residues forms stem-loop structure with UAU sequence (Figure 3C—left panel) indicating, that *MCL-1* mRNA is a novel potential target for RNase activity of MCPIP-1 (30). We then showed the binding of *MCL-1* transcript to MCPIP-1 protein by the immunoprecipitation method (Figure 3D). Moreover, we found that the identified sequence of human *MCL-1* mRNA resembles the murine *Mcl-1* mRNA fragment between 1,833 and 1,861 nt (NM_008562.3) and is conserved between other species (Figure 3C—right panel). Furthermore, ΔG values for *MCL-1* stem-loop structures were low in most species, in the range from −9.2 to −6.1 kcal/mol, indicating their stability (Figure 3C—right panel). Due to the homology of human and murine RNase-target sequences, we used neutrophils isolated from peritoneum of *control mutant* and transgenic *Mcpip-1-KO* animals and demonstrated that the level of mRNA and protein for *Mcl-1* and *Bcl2A1* (but not pro-apoptotic *Bid* and *Bim)* strongly depends on MCPIP-1 expression (Figure 3E with insert). The comparison of *Mcl-1/Bim* expression ratio shows vast predominance (15-times) of anti-apoptotic over pro-apoptotic transcripts in cells deprived of MCPIP-1 (Figure 3F). We showed a similar trend, albeit less significant in bone marrow neutrophils (Supplementary Figures 2A,B). Taking together, we revealed that MCPIP-1 controls the balance between anti- and pro-apoptotic proteins in neutrophils thus regulating their life-span.

### MCPIP-1 Dependent Cytoprotective Activity of GM-CSF on Neutrophils

**Figure 4:**
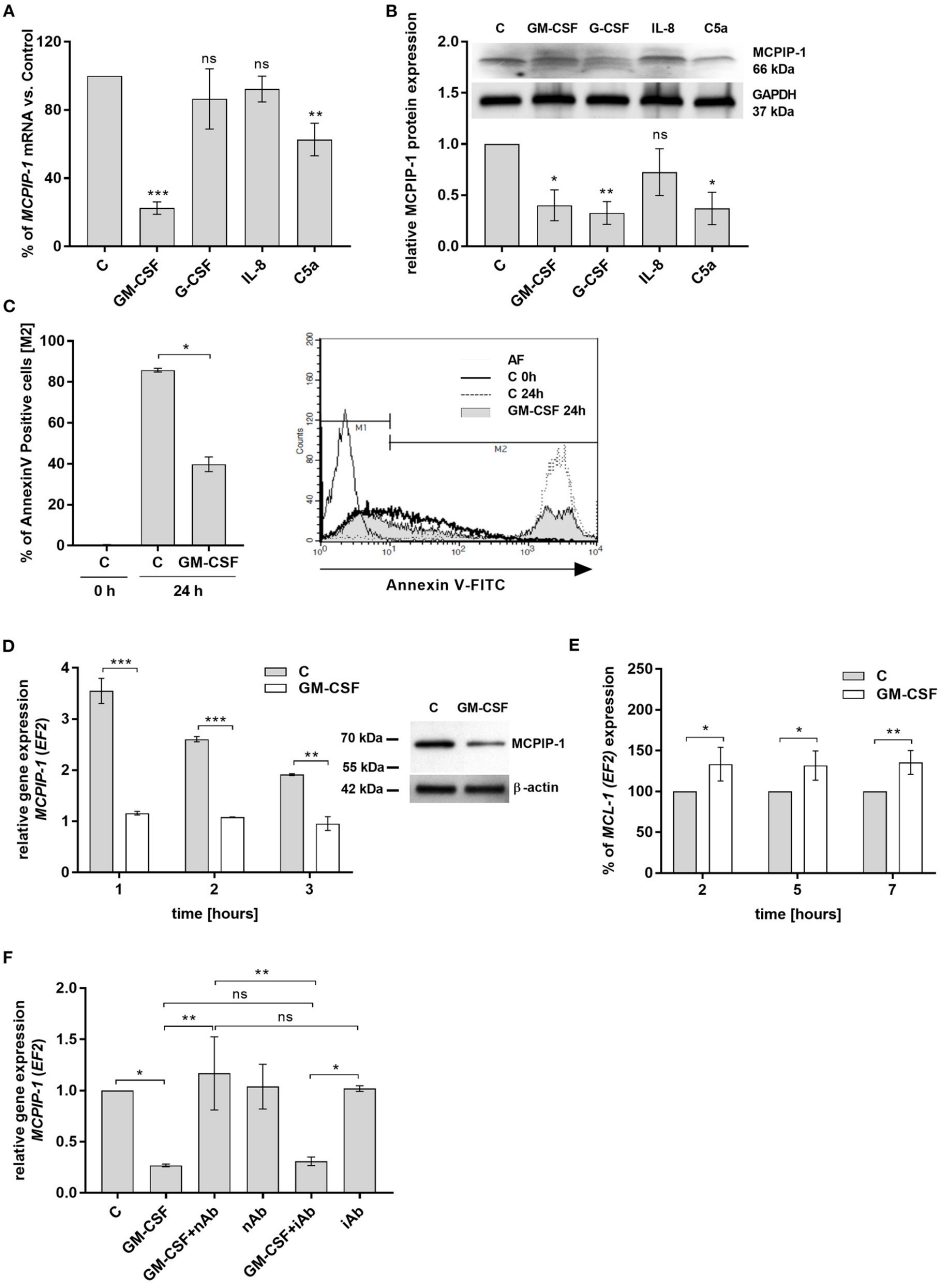
MCPIP-1 is engaged in the regulation of neutrophil life span by GM-CSF. **(A,B)** Freshly isolated human neutrophils were incubated with PBS or 20 ng/ml GM-CSF, 10 ng/ml G-CSF, 200 ng/ml IL-8, 2 μg/ml C5a for 2 or 4 h, respectively. **(A)** *MCPIP-1* was estimated 2 h post cells stimulation using qPCR. Data shows the percent of mean control values ± SD. *N* = 3–7 **(B)** The level of MCPIP-1 protein in the cells was determined 4 h post stimulation by Western blot. Densitometry analysis demonstrating MCPIP-1 protein fold change was normalized to GAPDH level. A representative result is depicted above. **(C)** Neutrophils were incubated in the presence of 20 ng/ml GM-CSF for 24 h. Cells were double-stained with Annexin V and PI and analyzed using flow cytometry. The histogram shows mean values ± SD; *n* = 3. A representative histogram depicts immunostaining analysis. **(D)** Kinetic changes of *MCPIP-1* mRNA in PMNs cultivated with or w/o GM-CSF. Data obtained from a representative donor was presented ± SEM (left). The level of MCPIP-1 protein after incubation of cells with GM-CSF for 3 h was determined by Western blot analysis (right). A representative result is depicted. **(E)** Kinetic changes of *MCL-1* mRNA in PMNs cultivated with or w/o GM-CSF. Data obtained from three independent donors presented as the percent of control ± SD. **(F)** Neutrophils were exposed for 1 h to GM-CSF previously incubated or not for 15 min with neutralizing or isotypic anti-GM-CSF antibodies. Control cells were treated with PBS or antibodies alone. After isolation of total RNA *MCPIP-1* was measured using qPCR. A representative result out of 3 was presented ± SEM. *p*-value of < 0.05 (*); *p*-value of < 0.01 (**); *p*-value of < 0.001 (***); ns for non-significant.

Spontaneous apoptosis of neutrophils can be affected by inflammatory mediators, like GM-CSF, G-CSF, C5a, or IL-8, which act in a cytoprotective manner (33–36). Therefore, we examined the influence of these molecules on MCPIP-1 expression. Our data showed that GM-CSF and C5a significantly decrease the level of *MCPIP-1* mRNA, albeit with the predominant effect exhibited by GM-CSF (Figure 4A). The effect was observed also on the protein level (Figure 4B) however we found the downregulation of MCPIP-1 also upon G-CSF stimulation (Figure 4B). Such observation corroborates with the reduction of spontaneous apoptosis of neutrophils in response to GM-CSF treatment (Figure 4C). Further experiments showed the downregulation of *MCPIP-1* mRNA and protein shortly post PMNs stimulation with GM-CSF (Figure 4D and insert). The above observation negatively correlates with *MCL-1* expression (Figure 4E). Next, we confirmed the finding using GM-CSF-neutralizing antibodies, which entirely restored *MCPIP-1* mRNA level (Figure 4F). Collectively, we revealed that MCPIP-1 modulates the cytoprotective activity of GM-CSF, which sheds new light on the poorly explained molecular mechanism of anti-apoptotic activity of GM-CSF.

### *MCPIP-1*-Mediated Neutrophils' Apoptosis Correlates With Reduced Expression of miRNAs Targeting MCPIP-1 Transcript

**Figure 5:**
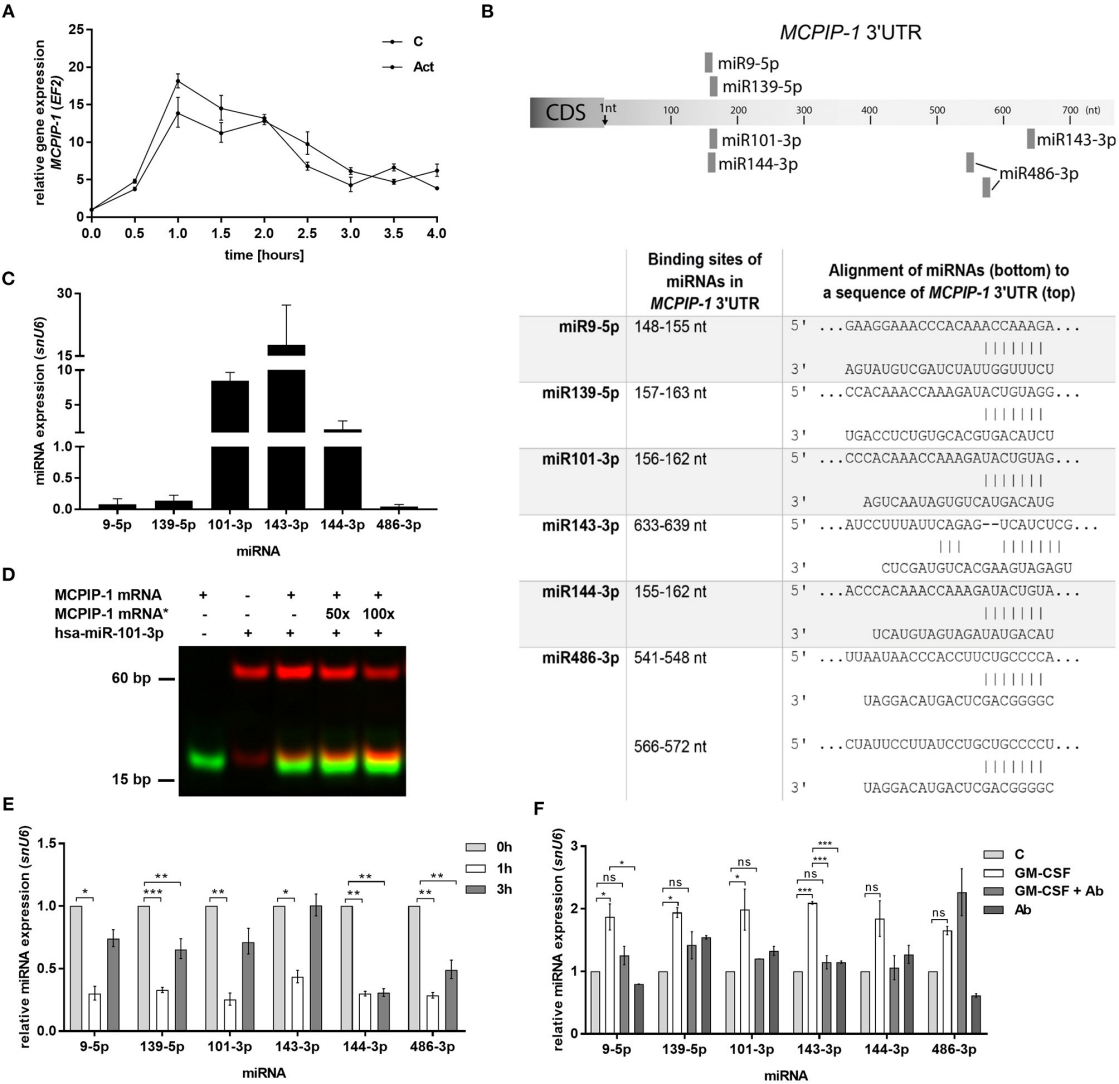
Increased stability of MCPIP-1 in neutrophils during apoptosis depends on miRNA depletion. **(A)** Freshly isolated human PMNs were preincubated for 1 h with 5 μg/ml of actinomycin D, then RNA was isolated and *MCPIP-1* determined using qPCR. Data represent the relative expression of *MCPIP-1* mRNA from one (out of 3) representative donor ± SEM. The level of *MCPIP-1* directly post PMNs isolation was taken as 1. C, control; Act, actinomycin D; **(B)** Identification of miRNAs binding sites in the *MCPIP-1* 3′UTR transcript. **(C)** The mean expression of 6 sequences of miRNA was determined using qPCR. ± SD, *n* = 3–4; **(D)** Binding 200 nM Atto488-labeled *MCPIP-1* oligonucleotide with 200 nM Cy5-labeled miR101-3p sequence was performed using EMSA assay. A representative result was presented. (*)-MCPIP-1 mRNA without dye **(E)** After indicated time post PMNs isolation from human blood the level of the miRNA expression was determined. Data represent results derived from a representative donor. Bars represent the relative miRNA expression to time 0 (*t*_0_ = 1) ± SEM. **(F)** Neutrophils were exposed for 2 h to GM-CSF previously incubated or not for 15 min with neutralizing anti-GM-CSF antibodies. Control cells were treated with PBS or antibodies alone. qPCR analysis was performed for the estimation of miRNA expression relative to PBS treated cells equal 1. A representative result out of 3 was presented ± SEM. *p*-value of < 0.05 (*); *p*-value of < 0.01 (**); *p*-value of < 0.001 (***); ns for non-significant.

In the next step, we examined the mechanism of rapid upregulation of *MCPIP-1* which proceeds apoptosis features (Figure 2C). Firstly, we excluded *de novo* synthesis as *MCPIP-1* mRNA expression level remains unchanged in the presence of actinomycin D (Figure 5A). Therefore, we focused on the miRNA analysis as one of the molecular pathways responsible for transcript stability. So far, only a few miRNA-binding sequences within the *MCPIP-1* transcript were identified, including miR9-5p- and miR139-5p-binding sites (37, 38). Our bioinformatics analysis pointed out several miRNAs, which possess sequences, that might regulate MCPIP-1 expression. We selected miR101-3p, miR143-3p, miR144-3p, and miR486-3p (Figure 5B) conserved between several species (Supplementary Figure 3). We found high expression of miR101-3p, miR143-3p, and miR144-3p compared to miR139-5p, miR9-5p, and miR486-3p (Figure 5C) in freshly isolated PMNs suggesting their role in the physiology of neutrophils. Moreover, for miR101-3p we confirmed binding to studied mRNA (Figure 5D). We found their rapid downregulation already 1 h post PMNs isolation (Figure 5E), while an exposition of neutrophils to GM-CSF caused transient upregulation of miR9-5p, miR139-5p, miR101-3p, and miR143-3p. This can be significantly limited by GM-CSF neutralizing antibodies suggesting the sophisticated interdependency between miRNAs/RNase and GM-CSF (Figure 5F). To sum up, the increased level of miRNAs that target MCPIP-1 affects its stability and may explain the prolonged neutrophil survival in the presence of GM-CSF. Taken together the obtained results revealed for the first time the phenomenon and the putative miR-dependent mechanism of pro-apoptotic function of MCPIP-1 in neutrophils.

## Discussion

MCPIP-1 is classified as a negative regulator and an anti-inflammatory molecule; however, its primary role was dedicated mainly to the inhibition of NFκB-dependent expression of proinflammatory cytokines. Here we revealed that the anti-inflammatory role of MCPIP-1 is much more comprehensive than previously suggested as it controls the accumulation of neutrophils in an inflammatory milieu. We evidenced that the protein serves as a potent regulator of neutrophil cell death *ex vivo*.

Previous reports indicated that some negative regulators of inflammation have a strong influence on neutrophils cell death. An example is SOCS3, which reduces the cytoprotective effect of G-CSF (39). On the other hand, SHP-1 recruitment to the Fas death receptor counteracts the impact of anti-apoptotic molecules (40) and tristetraprolin (TTP)-deficient granulocytes exhibit prolonged survival (41). Previous studies described the role of MCPIP-1 in the process of cell death, indicating its role in degradation of transcripts encoding anti-apoptotic proteins (13). Here, we observed a similar correlation. Additionally, we identified *MCL-1* mRNA as a novel yet unidentified potential target of MCPIP-1.

In our study, we analyzed the process of neutrophils' death *ex vivo*. However, it should be underlined that MCPIP-1 plays a pleiotropic role being a regulator of many genes. Therefore, we hypothesized that deficiency of MCPIP-1 can influence neutrophil mobilization from the bone marrow, attraction to the place of infection, bactericidal functions, turnover, or elimination from the inflammatory milieu. In our manuscript, we documented for example the inhibitory role of MCPIP-1 on chemokine secretion in the model of peritonitis. Taken together, advanced *in vivo* studies are needed to create the complete picture of the role of MCPIP-1 in the regulation of neutrophils' function to define the environmental factors and host molecules which could impact the function of MCPIP-1.

During inflammation cell death is modulated by a plethora of soluble mediators delaying the course of apoptosis (33–36). Our data revealed that they distinctively influence the expression of MCPIP-1 in neutrophils, with the strongest effect observed for GM-CSF. The GM-CSF-dependent mechanism of action promoting neutrophils' survival remains unsolved. Previous studies showed that GM-CSF prolonged neutrophil survival by induction of *de novo* synthesis of series mRNA and protein (42). Activation of Lyn kinase, Janus kinase/STAT, and PI3K, as well as regulation of MCL-1 and BAD proteins, were indicated as crucial players in survival mediated by GM-CSF (43–46). Described by us MCPIP-1-dependent MCL-1 regulation (putatively via stem-loop of *MCL-1* transcript) suggests the existence of a novel pathway, which modulates the cytoprotective activity of GM-CSF.

Some hypotheses explaining the mechanism of neutrophils' spontaneous apoptosis indicate that the initial step of this programmed cell death is a result of deprivation of cytoprotective cytokines in the microenvironment (47). Not to mention, decreased transcription and translation activities in mature neutrophils were also postulated to be involved in initiation of the neutrophil death process (48) as well as the balance between anti- and pro-apoptotic proteins, generation of ROS, and the release of intrinsic proteases (48, 49). For instance, the study of Pongracz et al. (50) showed the crucial role of PKC-δ kinase in this process. Other authors revealed that Akt deactivation is a key preliminary mediator of programmed cell death (31). They pointed out that the autocrine release of chemokines elicits PI3Kγ activation via G protein-coupled receptors and maintain Akt activity. In contrast, simultaneous accumulation of ROS in apoptotic neutrophils suppresses PI3Kγ activity, subsequent accumulation of their products, and consequently activation of Akt. Based on our results we proposed that the increased MCPIP-1 stability followed by its accumulation is an early event in the apoptosis process. We demonstrated that the high level of mRNA encoding MCPIP-1 could be regulated by microRNAs, which are known for their transcript- and translation-modulating properties. Besides already described miRNAs sequences (miR9 and miR139) we identified new, highly conservative miRs, which can bind to 3′UTR region of *MCPIP-1* mRNA. Importantly, unlike already described miRs, the new sequences (miR101, miR143, and miR144) display a significantly higher basal level of expression in neutrophils indicating a rush mechanism of action upon stimuli. The *MCPIP-1* transcript remains stable due to the rapid degradation of miRNA, which consequently affects the level of anti-apoptotic transcripts (MCPIP-1-dependent degradation). Our hypothesis is supported by studies showing that neutrophils exposed to GM-CSF exhibit delayed senescence and apoptosis foreshadowed by upregulation of some miRNA sequences (51). Our results confirmed that GM-CSF limits depletion of a majority of tested miRNA sequences, except miR144 and miR486. This leads us to the conclusion that the anti-apoptotic activity of GM-CSF is related to degradation of *MCPIP-1* transcript through induction of set of miRNA in PMNs in consequence promoting the survival of granulocytes. There are still some open questions, which have to be addressed in the future, such as the identification of the primary factor affecting miRNA level, as well as detailed regulation of GM-CSF-MCPIP-1 axis. Moreover, in presented study we have not provided direct evidence showing the binding of selected miRNAs to MCPIP-1 in neutrophils as the application of miRNA antagonists in this particular model will not show a desirable effect. It could be done using immortalized cell line characterized with the high level of studied miRNA treated with selected antimirs. Another method is design of the cell line with expression of MCPIP-1 with mutation in sequences which are recognized by studied miRNAs.

To sum up, here we show for the first time that MCPIP-1 is upregulated in dying neutrophils due to miRNA-dependent stabilization of its transcript and controls expression of anti-apoptotic genes at the early stage of apoptosis. This effect is counteracted by GM-CSF that downregulates MCPIP-1 and thus delaying apoptosis, which results in excessive accumulation of neutrophils and inflammatory tissue damage. Thus, the knowledge about the role of MCPIP-1 in PMNs' apoptosis may provide novel therapeutic targets for inflammatory diseases of neutrophil-based etiology.

## Data Availability Statement

The original contributions presented in the study are included in the article/Supplementary Material, further inquiries can be directed to the corresponding author/s.

## Ethics Statement

The animal study was reviewed and approved by the Institutional Animal Care and Use Committees (Jagiellonian University, Krakow, Poland; permit number: 228/2017; 92/2020).

## Author Contributions

ED and JK designed experiments and supervised data analysis: ED, MK, JK, DB, MH, ML, and MWa performed experiments with human and murine neutrophils and analyzed data: MWi performed bioinformatic analysis. ED and JK planned and supervised the project and prepared original draft. All authors commented on previous versions of the manuscript, read, and approved the final manuscript.

## Conflict of Interest

The authors declare that the research was conducted in the absence of any commercial or financial relationships that could be construed as a potential conflict of interest.
