# Supplementary material for: MCPIP-1 Restricts Inflammation via Promoting Apoptosis of Neutrophils
Source: Front Immunol. 2021 Feb 26;12:627922. doi: 10.3389/fimmu.2021.627922 (PMC7952515; doi:10.3389/fimmu.2021.627922)
Supplement: Supplementary file 1 [file Presentation_1.pdf]

Supplementary Material

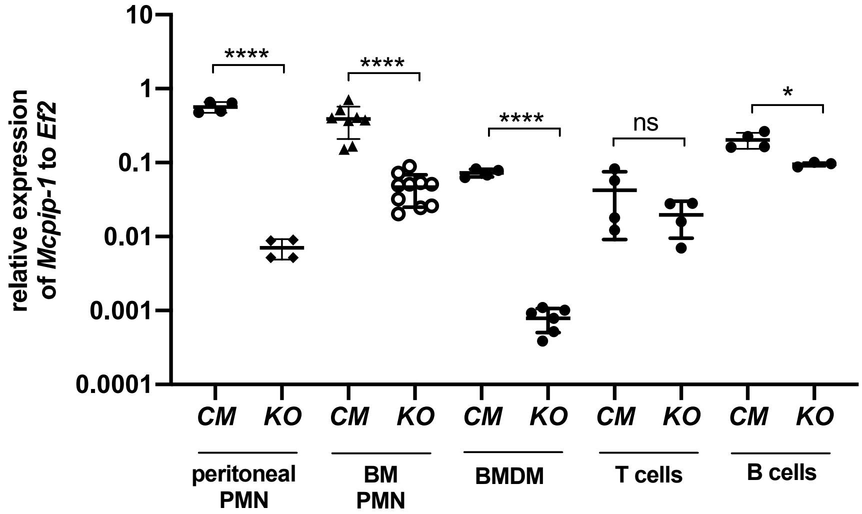

Figure S1. Selective silencing of *Mcpip-1* gene in phagocytes of MCPIP1-Flox-M-Lys-CRE mice. The level of *Mcpip-1* expression in neutrophils isolated from peritoneum of thioglycolate i.p. injected *Control Mutant (CM)* and *Mcpip-1-KO* (*KO*) mice, PMN derived from bone marrow, bone marrow derived macrophages (BMDMs), T and B lymphocytes isolated from the murine spleen was determined using Real-time PCR; the mean ± SEM; n=3-7

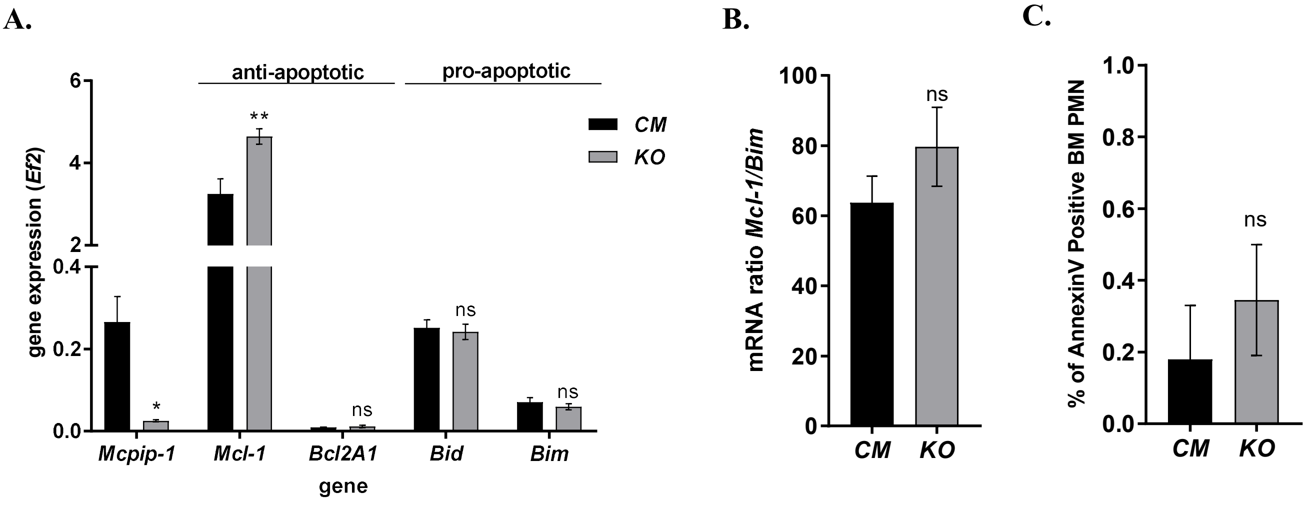

Figure S2. MCPIP-1 regulates *Mcl-1* in bone marrow neutrophils.
(A) Relative mean expression of apoptotic genes in murine neutrophils isolated from bone marrow of *Control Mutant* (*CM*) and *Mcpip-1-KO* (*KO*) mice. n=4 animals; **(B)** The graph represents a ratio between expression of *Mcl-1* and *Bim* mRNA in murine neutrophils isolated from bone marrow ± SEM. **(C)** Neutrophils were double-stained with Annexin V and PI and subjected to FACS. A representative result of Annexin V positive cells is shown with the mean ± SEM

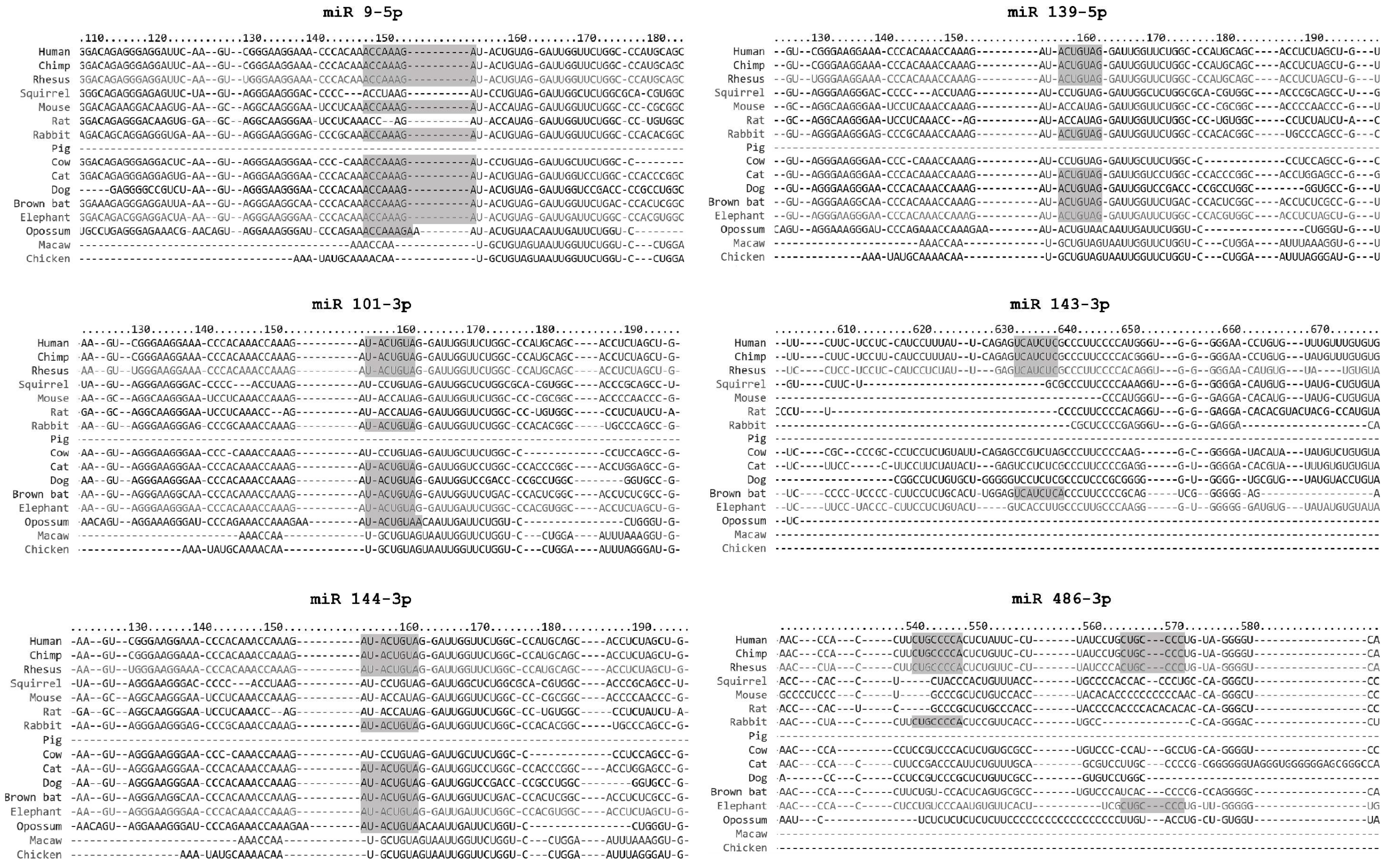

Figure S3. Alignment of predicted miRNAs seeds regions that match the sequence of human *MCPIP-1* 3'UTRs and its orthologs. Human - *Homo sapiens*; Chimp - *Pan troglodytes*; Rhesus - *Macaca mulatta*; Squirrel - *Spermophilus tridecemlineatus*; Mouse - *Mus musculus*; Rat - *Rattus norvegicus*; Rabbit - *Oryctolagus cuniculus*; Pig - *Sus scrofa*; Cow - *Bos taurus*; Cat – *Felis catus*, Dog - *Canis lupus familiaris*, Big brown bat – *Eptesicus fuscus*, Elephant - *Loxodonta africana*; Opossum - *Monodelphis domestica*, Scarlet macaw - *Ara macao*, Chicken - *Gallus gallus*.
